# Toxic epidermal necrolysis and acute kidney injury following co-trimoxazole rechallenge and voriconazole accumulation as an exacerbating cofactor: a case report

**DOI:** 10.3389/ftox.2026.1797771

**Published:** 2026-06-22

**Authors:** Yu-Dong Zhao, San-Lan Wu, Tao Zhou, Chen Shi, Wei-Jing Gong

**Affiliations:** 1 Department of Pharmacy, Union Hospital Tongji Medical College Huazhong University of Science and Technology, Wuhan, Hubei, China; 2 Department of Pharmacy, Yellow River Sanmenxia Hospital, Sanmenxia, China

**Keywords:** acute kidney injury, case report, co-trimoxazole, toxic epidermal necrolysis, voriconazole

## Abstract

**Background:**

Toxic epidermal necrolysis (TEN) is a life-threatening severe cutaneous adverse drug reaction with high mortality. Acute kidney injury (AKI) is a common complication of TEN, significantly increasing mortality risk. Immunocompromised patients frequently require combination therapy with voriconazole and co-trimoxazole for concurrent infections, which may lead to complex toxicities.

**Case Presentation:**

A 60-year-old male with nephrotic syndrome on long-term corticosteroids was diagnosed with *Aspergillus* and *Pneumocystis jirovecii* co-infection. He was initiated on voriconazole and co-trimoxazole on Day 6 and discharged on Day 9. Following a 20-day interruption of co-trimoxazole, he resumed the drug on Day 37 and developed widespread mucocutaneous erythema within hours. He presented on Day 40 with stage 2 AKI (eGFR 39 mL/min/1.73 m^2^) and severe systemic inflammation. Upon admission on Day 42, TEN was diagnosed with skin detachment >30% body surface area. Trough voriconazole level was 9.81 mg/L (therapeutic upper limit 5.5 mg/L). Pharmacogenetic testing revealed CYP2C19 *1/*2 genotype (intermediate metabolizer). ALDEN score was 9 for co-trimoxazole (“highly probable”) and 4 for voriconazole (“probable”). Renal replacement therapy was initiated on Day 49. Despite comprehensive treatment and transient improvement of skin lesions, the patient ultimately died on Day 63, 17 days after the initiation of hemodialysis. The precise cause of death could not be determined.

**Conclusion:**

Co-trimoxazole rechallenge strongly supports a causal link to TEN. AKI pathogenesis was multifactorial: primarily driven by TEN-associated systemic inflammation, with voriconazole accumulation due to CYP2C19 intermediate metabolism and drug interaction serving as a likely exacerbating co-factor. This case underscores the importance of therapeutic drug monitoring, pharmacogenetic testing, and integrated cross-organ surveillance when using high-risk antimicrobial combinations.

## 1 Introduction

The management of immunocompromised patients with complex pneumonias frequently requires antimicrobial combinations to cover diverse pathogens. Co-administration of voriconazole and co-trimoxazole is often employed for suspected *Aspergillus* and *Pneumocystis jirovecii* co-infections. Co-trimoxazole carries well-documented risks of life-threatening TEN, while voriconazole is predominantly associated with concentration-dependent neurological and hepatic toxicity (Dolton and McLachlan, 2014; Yan et al., 2025). When used concomitantly, severe adverse events such as TEN and AKI may emerge concurrently. AKI is not only a common complication of TEN but also an established prognostic factor for increased mortality (Hung et al., 2009; Lee et al., 2018; Rattanakaemakorn et al., 2022). This report presents a case of such concomitant toxicities, employing detailed clinical timeline analysis, pharmacogenetic testing, and therapeutic drug monitoring to delineate the distinct and potentially overlapping pathophysiological contributions.

## 2 Case presentation

A 60-year-old Chinese man with nephrotic syndrome on long-term corticosteroid therapy (methylprednisolone 30 mg daily) was admitted for severe pneumonia on April 11 (Day 0). Due to progressive hypoxemia and lack of response to 5 days of initial empirical antibiotic therapy with intravenous piperacillin-tazobactam (4.5 g every 8 h), bronchoscopy with bronchoalveolar lavage was performed. Co-infection with *Aspergillus* and *P. jirovecii* was microbiologically confirmed by metagenomic next-generation sequencing of bronchoalveolar lavage fluid. Antifungal and antimicrobial therapy was initiated on Day 6 with intravenous voriconazole (loading dose 400 mg every 12 h, then 200 mg every 12 h) and oral co-trimoxazole (1,200 mg/240 mg every 8 h). Concomitant medications included pantoprazole, and linagliptin. The patient was discharged on Day 9.

At a follow-up on Day 25, renal function was preserved (estimated glomerular filtration rate [eGFR] 143.37 mL/min/1.73 m^2^), but liver enzymes were elevated. The patient reported unintentional interruption of co-trimoxazole from Day 17 to Day 36 due to medication exhaustion. He had continued oral voriconazole (200 mg every 12 h) without dose adjustment. The patient was seen in outpatient clinics on Day 28 and Day 36 for routine follow-up, during which no overt symptoms of renal impairment were reported, though renal function testing was not performed. Within a short time after resuming co-trimoxazole on Day 37, he developed widespread painful mucocutaneous erythema. He presented to the emergency department on Day 40. Voriconazole was discontinued, and new-onset acute kidney injury (AKI) was documented (eGFR 39.13 mL/min/1.73 m^2^), meeting the KDIGO stage 2 criteria (Kellum and Lameire, 2013). Laboratory tests on Day 40–41 revealed a profound systemic inflammatory state: leukocytosis (20.5 × 10^9^/L) with neutrophilia (88%), C-reactive protein (146.18 mg/L), hypoalbuminemia (18.8 g/L), soluble CD25 (9616 U/m), and ferritin (1,085.8 μg/L).

**FIGURE 1:**
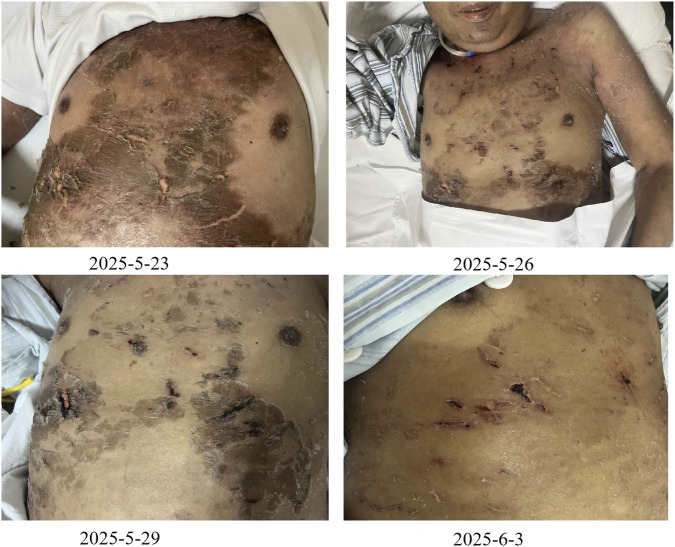
Evolution of chest epidermolysis from admission to resolution.

Upon admission on Day 42, toxic epidermal necrolysis (TEN) was diagnosed clinically, supported by a typical Nikolsky sign and skin detachment affecting >30% of the body surface area (Seo et al., 2021). Trough voriconazole concentration was 9.81 mg/L, exceeding the therapeutic upper limit of 5.5 mg/L (Takesue et al., 2022). This sample, collected 30 min prior to the next scheduled dose, represented a true trough concentration. Renal function had further declined, with an eGFR of 30.15 mL/min/1.73 m^2^ on Day 42, and deteriorated to 15.12 mL/min/1.73 m^2^ by Day 46. Urinalysis showed mild proteinuria (1+) without hematuria or evidence of crystalluria. Peripheral eosinophil counts, obtained from complete blood count, were normal throughout the clinical course. All systemic medications were discontinued. Dermatological management included meticulous wound care with non-adherent dressings, supplemented by topical recombinant bovine basic fibroblast growth factor gel and vitamin E cream twice daily from Day 43. Strict fluid and electrolyte management was maintained. Nutritional support was provided orally, with emphasis on high-protein intake to address the hypercatabolic state. Supportive care included intravenous human serum albumin (10 g daily) for severe hypoalbuminemia, and intravenous magnesium isoglycyrrhizinate (200 mg daily) and reduced glutathione (2.4 g daily) for progressive cholestatic liver injury, both initiated on Day 48. Due to progressive oliguria and worsening azotemia (eGFR 12.86 mL/min/1.73 m^2^ on Day 47), intermittent hemodialysis was initiated on May 28. Renal replacement therapy was continued, with hemodialysis sessions on Day 47, Day 49, Day 51, and Day 53. eGFR was 11.37 mL/min/1.73 m^2^ on Day 49, rising to 14.58 and 15.33 mL/min/1.73 m^2^ on Day 52 and Day 53, respectively, indicating early signs of recovery while on dialysis. On Day 54, the patient was discharged to a local township hospital for continued hemodialysis. However, his subsequent course was complicated by intermittent fevers, and he received dialysis on Day 56, Day 59, Day 61, and Day 63. Tragically, the patient died suddenly at 7 p.m. on Day 63, shortly after completing a hemodialysis session. The precise cause of death could not be determined, but potential contributors include an acute cardiovascular event, sepsis, or a late complication of TEN-associated systemic inflammation. No autopsy was performed. The skin lesions gradually improved thereafter (Figure 1).

Markers of the systemic inflammatory response remained strikingly elevated throughout the following week, including C-reactive protein (consistently >80 mg/L), procalcitonin (peaking at 4.78 ng/mL), ferritin (>2000 μg/L), and soluble CD25 (3073 U/mL). The patient developed significant cholestatic liver injury, evidenced by progressive elevations in alkaline phosphatase (up to 686 U/L), γ-glutamyl transferase (up to 521 U/L), and bilirubin (total 60.4 μmol/L, direct 44.2 μmol/L). A consumptive coagulopathy was also evident, with persistent elevations in D-dimer (up to 8.56 mg/L FEU) and intermittent prolongation of clotting times. Comprehensive infectious workups, including serial blood cultures and extensive respiratory pathogen PCR panels, were negative, supporting the non-infectious, inflammatory nature of his deterioration.

Pharmacogenetic analysis of the CYP2C19 gene was performed using the Sequenom MassARRAY System, which tested for the common variant alleles *2 (c.681G>A), *3 (c.636G>A), and *17 (c.-806C>T). The results identified a *1/*2 genotype, consistent with an intermediate metabolizer phenotype. Testing for the SCAR-associated alleles HLA-B*15:02 and HLA-B*58:01 was negative. Delayed clearance of voriconazole was evident, with a persistent level of 6.32 mg/L measured on Day 47.

**FIGURE 2:**
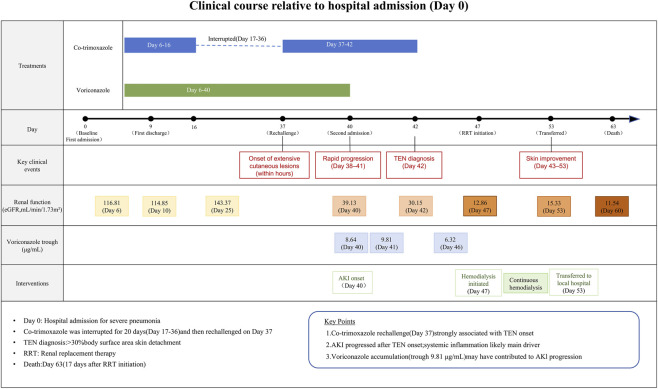
Timeline of drug exposure, clinical events, and laboratory findings.

The temporal relationship between drug therapy, the onset of TEN and AKI, key laboratory findings, and clinical interventions is summarized in Figure 2 and Supplementary Table 1. This study was approved by the Ethics Committee of the Union Hospital, Tongji Medical College, Huazhong University of Science and Technology ([2025] shen (1,397–01)).

## 3 Discussion

### 3.1 Causality of TEN: co-trimoxazole rechallenge as the principal trigger

The development of TEN in this patient is most convincingly attributed to co-trimoxazole. The rapid onset of symptoms within hours of resuming the drug after a 20-day hiatus exemplifies a classic rechallenge phenomenon, reflecting a recall response from pre-sensitized, drug-specific T cells. The ALDEN algorithm assigned co-trimoxazole a score of 9 (“highly probable”), driven by the suggestive delay from rechallenge to onset, the positive rechallenge for both the disease and the drug, and the drug’s well-established association with TEN (Supplementary Table 2) (Sassolas et al., 2010). In contrast, voriconazole received a score of 4 (“probable”). This pronounced score difference, combined with the unequivocal positive rechallenge, firmly establishes co-trimoxazole as the principal drug responsible for TEN and should not be interpreted as suggesting dual primary causality.

Testing for HLA-B15:02 and HLA-B58:01 was negative. This is not unexpected, as these alleles are primarily associated with carbamazepine- and allopurinol-induced SCARs, respectively, and their absence does not meaningfully exclude genetic susceptibility to co-trimoxazole hypersensitivity. Emerging evidence points to distinct alleles—such as HLA-B13:01 in Southeast Asian populations and HLA-B44:03 in European populations—as more relevant risk factors for co-trimoxazole-induced SCARs (Nakkam et al., 2022; Li et al., 2025). These were not tested, representing a limitation. The negative results thus indicate only that the reaction did not proceed via the specific pathways tested, highlighting the drug-specific and population-stratified nature of immunogenetic risk in SCARs.

### 3.2 Causality of AKI: TEN-driven systemic inflammation as the primary driver

Attributing the AKI to a single agent is challenging, but the evidence converges on a clear hierarchy. The temporal sequence strongly positions TEN as the primary driver: renal function was normal on May 6 (eGFR 143 mL/min/1.73 m^2^), skin manifestations began on May 18, and significant AKI (KDIGO stage 2) was documented by May 21. This rapid progression aligns with the well-recognized phenomenon of TEN-induced multi-organ injury, where a systemic “cytokine storm,” hemodynamic instability, and potential cross-reactivity of drug-specific T cells with renal antigens contribute to acute tubular damage (Hsu, 2001; Spanou et al., 2006; Hung et al., 2009).

Voriconazole accumulation is best interpreted as an exacerbating co-factor. The patient was a CYP2C19 intermediate metabolizer (*1/*2 genotype, a phenotype known to reduce voriconazole clearance significantly (Mikus et al., 2006). This was compounded by co-administration of pantoprazole, a CYP2C19 inhibitor that further elevates voriconazole exposure, especially in carriers of reduced-function alleles (Mafuru et al., 2021). The measured trough concentration of 9.81 mg/L (sampled 30 min before the next scheduled dose)—nearly twice the recommended upper limit of 5.5 mg/L—confirms supratherapeutic accumulation. Furthermore, the patient’s profound systemic inflammation (CRP >146 mg/L) likely suppressed CYP2C19 activity through inflammation-driven phenoconversion. As previously reported, patients can be phenoconverted to a lower metabolizer phenotype irrespective of CYP2C19 genotype when CRP exceeds 50 mg/L (Klomp et al., 2024), and this effect is particularly pronounced in intermediate and rapid metabolizers (Klomp et al., 2026). Thus, in this intermediate metabolizer patient, inflammation likely resulted in a functional shift to a lower metabolizer phenotype, contributing to impaired voriconazole clearance. While voriconazole-associated nephrotoxicity has been reported, the absence of typical signs of acute interstitial nephritis (normal eosinophil counts, bland urinalysis) suggests that its contribution was likely that of a superimposed toxic stressor on kidneys already rendered vulnerable by TEN-driven inflammation, rather than a primary nephrotoxic insult. Although co-trimoxazole can cause AKI through crystalluria or interstitial nephritis, the absence of relevant clinical signs and the overwhelming evidence of systemic inflammation make it a less immediate contributor in this specific presentation.

### 3.3 Pathophysiological interplay and clinical implications

The co-occurrence of TEN and AKI in this case propagated a synergistic vicious cycle. TEN-driven epidermal destruction and systemic inflammation created the substrate for renal injury, while the onset of AKI—itself a marker of disease severity and a predictor of mortality in TEN—likely altered drug clearance and amplified the toxic stress from accumulated voriconazole (Rattanakaemakorn et al., 2022). The reactive oxidative metabolites of sulfamethoxazole, the sulfonamide component of co-trimoxazole, may have further contributed through hapten formation and immune activation, aligning with the pharmacological interaction concept of T-cell activation in SCARs (Pichler et al., 2015; Bellón, 2019).

This case yields three actionable clinical imperatives. First, a history of prior exposure to high-risk drugs such as co-trimoxazole mandates extreme caution regarding re-exposure. If re-challenge is unavoidable, it should occur only with explicit informed consent and intensive monitoring. Second, preemptive therapeutic drug monitoring for voriconazole is essential when co-administered with CYP2C19 inhibitors or in patients with suspected altered metabolism; the goal is to maintain trough concentrations within 1–5.5 mg/L, with the first trough obtained 2–5 days after initiation. Third, the interplay between cutaneous and renal toxicity demands integrated cross-organ surveillance: a new-onset rash in a patient receiving multiple high-risk medications warrants a structured “toxicity alert bundle,” comprising immediate medication review, urgent dermatology consultation, comprehensive laboratory evaluation including renal function, review of drug levels, and hemodynamic assessment. Conversely, unexplained renal decline should prompt an immediate skin examination. This bidirectional vigilance is essential for the early detection and mitigation of synergistic toxicity cycles, which, as this case tragically illustrates, can culminate in a fatal outcome despite intensive supportive care.

### 3.4 Limitations

Our analysis has several limitations. First, renal biopsy was not performed due to the patient’s critical condition, precluding definitive histopathological confirmation of the AKI etiology. Second, we cannot exclude contributions from the underlying nephrotic syndrome or other unmeasured confounders. Third, a synergistic nephrotoxic effect of co-trimoxazole and voriconazole cannot be ruled out. Fourth, while the ALDEN score strongly implicates co-trimoxazole for TEN, definitive proof of causality in multi-factorial cases remains challenging. Fifth, there was a gap in renal function monitoring between Day 25 and Day 40, although the patient was clinically asymptomatic during this period. Finally, the lack of autopsy data limits our understanding of the precise cause of death.

## 4 Conclusion

This report establishes a high-probability causal link between co-trimoxazole rechallenge and TEN, as robustly quantified by a high ALDEN score of 9 (highly probable). The pathogenesis of the concomitant AKI was multifactorial. It was most directly aligned with the systemic inflammatory effects of TEN, a well-documented complication. This primary insult was likely exacerbated by supratherapeutic exposure to voriconazole, resulting from CYP2C19 intermediate metabolism and a pharmacokinetic interaction. While a direct contribution from sulfamethoxazole toxicity could not be ruled out, the overwhelming evidence of systemic inflammation positioned it as a less immediate driver in this specific presentation. This case underscores three critical imperatives for managing high-risk antimicrobial therapy: vigilant therapeutic drug monitoring, preemptive pharmacogenetic profiling, and integrated cross-organ safety surveillance. The fatal outcome, despite transient dermatological improvement, highlights the lethality of TEN complicated by severe AKI and reinforces that early vigilance must translate into aggressive, coordinated intervention to prevent irreversible progression.

## Data Availability

The original contributions presented in the study are included in the article/Supplementary Material, further inquiries can be directed to the corresponding authors.
