# Supplementary material for: Toxic epidermal necrolysis and acute kidney injury following co-trimoxazole rechallenge and voriconazole accumulation as an exacerbating cofactor: a case report
Source: Front Toxicol. 2026 Jun 22;8:1797771. doi: 10.3389/ftox.2026.1797771 (PMC13333233; doi:10.3389/ftox.2026.1797771)
Supplement: Supplementary file 1 [file Table1.docx]

**Supplemental Table 1. Main therapeutic medications during hospitalization**

| Medication (INN/Generic Name) | Dosage | Route of Administration | Frequency | Administration Period |
| --- | --- | --- | --- | --- |
| Voriconazole Tablets | 200mg | Oral | Every 12 hours | Day 6 to Day 40 |
| Co-trimoxazole (Sulfamethoxazole/Trimethoprim) Tablets | 3 tablets | Oral | Three times daily | Day 6 to Day 17; Day 37 to Day 42 |
| Bicyclol Tablets | 50mg | Oral | Three times daily | Day 34 to Day 42; Day 48 to Day 53 |
| Pantoprazole Tablets | 40mg | Oral | Once daily | Day -32 to Day 42 |
| Linagliptin Tablets | 5mg | Oral | Once daily | Day -32 to Day 42 |
| Methylprednisolone Tablets | 30mg | Oral | Once daily | Day -32 to Day 42 |
| Calcitriol Capsules | 0.25μg | Oral | Once daily | Day -32 to Day 42 |
| Calcium and Vitamin D Chewable Tablets | 0.6g | Oral | Once daily | Day -32 to Day 42 |
| Recombinant Bovine Basic Fibroblast Growth Factor (rb-bFGF) | 1g | Topical | Twice daily | Day 43 to Day 53 |
| Vitamin E Cream | 1 g | Topical | Twice daily | Day 43 to Day 53 |
| Magnesium Isoglycyrrhizinate Injection | 200mg | IV infusion | Once daily | Day 48 to Day 53 |
| Reduced Glutathione for Injection | 2.4g | IV infusion | Once daily | Day 48 to Day 53 |
| Human Serum Albumin Injection | 10g | IV infusion | Once daily | Day 48 to Day 53 |

**Supplemental Table 2. ALDEN (Algorithm of Drug Causality for Epidermal Necrolysis) Scores for Each Suspect Drug**

| Criterion | Values | Rules to apply | Voriconazole Tablets | Co-trimoxazole Tablets | Bicyclol Tablets | Pantoprazole Tablets | Linagliptin Tablets | Methylprednisolone Tablets | Calcitriol Capsules | Calcium and Vitamin D Chewable Tablets |  |
| --- | --- | --- | --- | --- | --- | --- | --- | --- | --- | --- | --- |
| Delay from initial drug component intake to onset of reaction (index day) | Suggestive +3 | From 5 to 28 days |  | 3 |  |  |  |  |  |  | |
|  | Compatible+2 | From 29 to 56 days | 2 |  |  |  |  |  |  |  | |
|  | Likely +1 | From 1 to 4 days |  |  | 1 |  |  |  |  |  | |
|  | Unlikely -1 | >56 Days |  |  |  | -1 | -1 | -1 | -1 | -1 | |
|  | Excluded -3 | Drug started on or after the index day |  |  |  |  |  |  |  |  | |
|  |  | In case of previous reaction to the same drug,only changes for: Suggestive:+3:from 1 to 4 days Likely:+1:from 5to 56 days |  |  |  |  |  |  |  |  | |
| Drug present in the body on index day | Definite 0 | Drug continued up to index day or stopped at a time point less than five times the elimination half-life&before the index day | 0 | 0 | 0 | 0 | 0 | 0 | 0 | 0 | |
|  | Doubtful -1 | Drug stopped at a time point prior to the index day by more than five times the elimination half-life&but liver or kidney function alterations or suspected drug interactions are present |  |  |  |  |  |  |  |  | |
|  | Excluded -3 | Drug stopped at a time point prior to the index day by more than five times the elimination half-life,without liver or kidney function alterations or suspected drug interactions |  |  |  |  |  |  |  |  | |
| Prechallenge/rechallenge | Positive specific for disease and drug: 4 | SJS/TEN after use of same drug |  | 4 |  |  |  |  |  |  | |
|  | Positive specific for disease or drug: 2 | SJS/TEN after use of similar'drug or other reaction with same drug |  |  |  |  |  |  |  |  | |
|  | Positive unspecific:1 | Other reaction after use of similar drug |  |  |  |  |  |  |  |  | |
|  | Not done/ unknown: 0 | No known previous exposure to this drug | 0 |  | 0 | 0 | 0 | 0 | 0 | 0 | |
|  | Negative: -2 | Exposure to this drug without any reaction(before or after reaction) |  |  |  |  |  |  |  |  | |
| Dechallenge | Neutral 0 | Drug stopped(or unknown) | 0 | 0 | 0 | 0 | 0 | 0 | 0 | 0 | |
|  | Negative -2 | Drug continued without harm |  |  |  |  |  |  |  |  | |
| Type of drug (notoriety) | Strongly associated 3 | Drug of the"high-risk"list according to previous case-control studies | 3 | 3 |  |  | 3 | 3 |  |  | |
|  | Associated 2 | Drug with definite but lower risk according to previous case-control studies |  |  |  |  |  |  |  |  | |
|  | Suspected 1 | Several previous reports, ambiguous epidemiology results (drug “under surveillance”) |  |  |  |  |  |  |  |  | |
|  | Unknown 0 | All other drugs including newly released ones |  |  | 0 | 0 |  |  | 0 | 0 | |
|  | Not suspected -1 | No evidence of association from previous epidemiology study with sufficient number of exposed controls |  |  |  |  |  |  |  |  | |
|  |  | Intermediate score = total of all previous criteria | 5 | 10 | 1 | -1 | 2 | 2 | -1 | -1 | |
| Other cause | Possible -1 | Rank all drugs from highest to lowest intermediate score |  |  |  |  |  |  |  |  | |
|  |  | If at least one has an intermediate score>3,subtract 1 point from the score of each of the other drugs taken by the patient (another cause is more likely) | -1 | -1 | -1 | -1 | -1 | -1 | -1 | -1 | |
| Final score-12 to 10 | | | 4 | 9 | 0 | -2 | 1 | 1 | -2 | -2 | |

<0, Very unlikely; 0–1, unlikely; 2–3, possible; 4–5, probable; ≥6, very probable
